# Injury to the Perineum Puncture of the Bladder above the Pubes

**Published:** 1856-03

**Authors:** George M. Fox

**Affiliations:** Brush Hill, Ill.


ARTICLE II.—*Injury to the Perineum puncture of the Bladder above the Pubes.* Reported by Dr. GEORGE M. FOX, of Brush Hill, Ill.

Aug. 6th, 1855 ; was called to Lyons to attend a boy by the name of DRUMMOND, 13 years of age, who had received an injury of the perineum by sliding from a hay mow, and alighting on the edge of a barrel, one foot going inside the other out. I found him with the scrotum considerably swollen, and somewhat ecchymosed ; he had received the injury the evening previous to my being called. He did not experience much pain when he kept quiet. Since receiving the injury he had passed a small quantity of urine with some pain. I ordered the injured part to be kept constantly wet with the following lotion : ℞ plumb. acet. ʒ x., tinct. opi. ℥ i, aqua. ℥ iv., to be kept cool with the addition of bits of ice, and applied often. Aug. 7th, he was much the same as yesterday. Ordered a laxative, and local treatment continued ; urine was passed with some pain. Aug. 8th, 9th, and 10th; remained much the same, the urine was passed with some difficulty, yet, passed off so as to cause no very unpleasant symptoms—local treatment continued. Aug. 11th, found him suffering from retention of urine, the bladder was moderately distended, with considerable pain and tenderness. I undertook to pass a catheter, and now, for the first time, discovered that the urethra was ruptured in the perineum, and that severely; as near as I could judge all efforts to pass a catheter were unavailing; something must be done. I dispatched a messenger for Dr. JOHNSON, of Chicago. The bladder must be emptied in some manner, and I did not like to hazard an operation without counsel, at any rate. He did not arrive until next morning. By this time, urinary infiltration had taken place to considerable extent, through the perineum and scrotum. He tried to introduce a catheter, but with no better success than myself. We then decided to puncture the bladder above the pubes, and Dr. JOHNSON proceeded to perform the operation in the usual manner. Canula was allowed to remain in the bladder until such time as we could succeed in introducing a catheter through the natural passage. Aug. 13th; patient doing well, had rested well through the night, the lower part of the scrotum beginning to show some appearance of sloughing; continued the same local application with morph. sulph. as an anodyne to relieve pain and procure sleep. From this time up to the 23d, there was no unpleasant symptoms followed the operation; but the lower part of the scrotum, and a portion of the perineum, had sloughed entirely away, leaving the testicles bare, and revealing the rupture of the urethra, nearly two inches of which sloughed entirely away. He had had a slight attack of diarrhœa which was rather troublesome, but yielded to the usual remedies. The parts seemed to manifest a disposition to heal rapidly. I now tried in vain to introduce a catheter through the natural passage, but all efforts were unavailing. Aug. 19th, I again called Dr. JOHNSON, in consultation. He succeeded better than I had done, but in a different manner. He passed, first, a small sized catheter through the opening above the pubes into the bladder, and then passing it out through the urethra into the perineum, then by bringing a second catheter in contact with this, the first was withdrawn, while the second was introduced; the canula above the pubes having been first withdrawn. The aperture healed rapidly; the urine was discharged through this catheter for a day or two, when it became displaced by accident. The urine was now discharged through the aperture in the perineum. Things remained in this shape until Aug. 28th; all efforts to introduce a catheter being unavailing. When, on trying to introduce a gum elastic catheter, the catheter passed beneath the bladder, and between that and the rectum, I felt something yield to the pressure I was making, and supposed that the catheter had entered the bladder, but it was only for a moment, for a large quantity of dark colored and very offensive smelling pus flowed through the instrument. On turning him on his side, nearly a half pint was discharged. I removed the catheter, and from this time to Sept. 5th, the abscess discharged profusely, the urine still passed through the perineum. About this time he had a severe attack of diarrhœa; this, together with the discharge from the perineum, and the abscess between the bladder and rectum, (for such it undoubtedly was,) debilitated him very much, he being reduced to a mere skeleton. At this time the opening in the perineum was healing rapidly, and something must be done to prevent the urethra from closing up entire, and the formation of a urinary fistulæ in the perineum. I finally succeeded in passing a gum elastic catheter after much trouble, in the following manner: I first passed the catheter through the natural passage, and at the aperture in the perineum, then doubling it on itself passed it into the bladder. In the mean time I had given him as nutritious a diet as he would bear, and procured rest by opiates. From this time the discharge from the abscess became rapidly less. Sept. 12th; the wound in the perineum still doing well. I can now pass a silver catheter, but with some trouble. From this time until Oct. 1st, he improved rapidly; the wound in the perineum had closed, and he passed urine through the natural passage as well as ever. He looks well, having regained his flesh and strength. Up to this time I had kept a catheter in the urethra most of the time, but have not used it since, and now at this time, Dec. 1st, he is as well as ever, and has had no symptoms of stricture or anything of the kind, and all that is left of the injury is a large cicatrix; more than half of the scrotum being entirely gone.

